# JESREC score, metabolic factors, and pulmonary dysfunction: analysis among adult patients with chronic rhinosinusitis

**DOI:** 10.3389/fmed.2026.1859696

**Published:** 2026-09-11

**Authors:** Ling Zhang, Haixiang Xue, Lei Xi, Jianqiang You

**Affiliations:** 1 Department of Otorhinolaryngology, The First People’s Hospital of Changzhou, The Third Affiliated Hospital of Soochow University, Soochow University, Changzhou, China; 2 Department of Endocrine and Metabolic Diseases, The Second People’s Hospital of Changzhou, The Third Affiliated Hospital of Nanjing Medical University, Nanjing Medical University, Changzhou, China

**Keywords:** chronic rhinosinusitis, JESREC score, mediation analysis, metabolic factors, pulmonary dysfunction

## Abstract

**Background:**

Chronic Rhinosinusitis (CRS), particularly its eosinophilic endotype (ECRS), is frequently associated with comorbid lower airway diseases. The JESREC score is a clinical tool for predicting ECRS. This study aimed to evaluate the association between the JESREC score and pulmonary dysfunction in adult CRS patients and to explore the potential role of metabolic factors.

**Methods:**

Patients were categorized based on pulmonary function tests and multivariate logistic regression analyses were performed to identify associated factors for overall pulmonary dysfunction and its restrictive and obstructive subtypes. The discriminative ability of the JESREC score was evaluated using Receiver Operating Characteristic (ROC) curve analysis. Furthermore, mediation models were constructed to figure out the mediating role of metabolic factors (BMI, blood pressure, fasting blood glucose and serum lipidomic profiles).

**Results:**

A total of 143 patients were included in the final analysis. The JESREC score was significantly higher in patients with pulmonary dysfunction (8.6 vs. 5.0, *P* < 0.001). Multivariate analysis confirmed the JESREC score (OR, 1.24, 95% CI, 1.12–1.37) and ECRS (5.17, 2.05–13.09) as associated factors for pulmonary dysfunction, including both restrictive and obstructive subtypes. The ROC curve showed an AUC of 0.729, with an optimal JESREC cutoff of 4.5. Mediation analysis indicated that systolic blood pressure, diastolic blood pressure, triglycerides, and HDL-C significantly mediated 6.4%, 4.5%, 8.4%, and 6.5% of the total effect, respectively.

**Conclusions:**

JESREC score was independently associated with pulmonary dysfunction in CRS patients, and metabolic factors may statistically mediate part of this association, suggesting a potential pathway. Furthermore, patients with JESREC score ≥ 5 may warrant special attention to pulmonary function.

## Introduction

Chronic rhinosinusitis (CRS) is a common and heterogeneous inflammatory disease of nasal sinuses, which seriously affects the quality of life of patients and imposes a huge burden on social economy (1). Beyond the local nasal symptoms, CRS is increasingly recognized as a systemic inflammatory disorder frequently associated with lower respiratory diseases, such as asthma and chronic obstructive pulmonary disease (COPD), supporting the concept of a “unified airway” (2, 3). Among them, the subtype of eosinophilic chronic rhinosinusitis (ECRS) has a more severe phenotype and poor therapeutic effect, which is often closely related to pulmonary disease (4–6).

The Japan Epidemiological Survey of Refractory Eosinophilic Chronic Rhinosinusitis (JESREC) scoring system has been developed as a practical clinical tool to distinguish different CRS types based on clinical factors and computed tomography findings (7). Previous studies have confirmed that while the utility of the JESREC score in forecasting CRS severity, taste and olfactory functions, and surgical outcomes has been documented, its relevance to pulmonary dysfunction has received scant attention, and no threshold value has been established to aid in clinical risk stratification (8–10). In addition, epidemiological studies have demonstrated a concurrent rise in the incidence of CRS, pulmonary dysfunction, and metabolic disorders (including hypertension, diabetes, and obesity) over the past two decades, although the underlying mechanisms linking these conditions remain largely unknown (11–13). The NHANES study showed a significant correlation between CRS and COPD, with CD163 and C3 as potential core targets (14). Pulmonary function impairment was associated with the extent of inflammatory lesions among Chinese adult CRS patients (15).

Based on these observations, our study was to dissect the complex relationship between the JESREC score, metabolic factors, and pulmonary dysfunction in adult CRS patients, and has three main objectives. First, to definitively establish the association between the JESREC score and pulmonary dysfunction among adult CRS patients; second, to investigate whether metabolic factors statistically mediate this relationship; and third, to evaluate the discriminative ability of the JESREC score and determine its optimal cutoff value for screening pulmonary dysfunction. Elucidating this potential pathophysiological pathway will provide novel insights into the mechanisms underpinning the unified airway and may reveal integrated management strategies.

## Materials and methods

### Study population and design

This cross-sectional study was conducted at the Third Affiliated Hospital of Soochow University between March 2021 and September 2022 and participants were patients diagnosed with CRS in the department of otolaryngology according to EPOS 2012 diagnostic criteria (16). As described in our previous studies (9, 17), patients were enrolled according to strict criteria. In this study, after excluding patients with missing pulmonary function data, history of asthma, COPD and other lower respiratory diseases, a total of 143 patients were included in the final analysis. The Ethics Committee of The Third Affiliated Hospital of Soochow University reviewed and approved the study protocol (2022CL070). A waiver of informed consent was obtained because the study was based on anonymous data and was non-interventional in nature.

### Definition of JESREC score and ECRS

The JESREC score was calculated for each patient based on established criteria, which include factors such as bilateral lesion, nasal polyps, and the predominance of ethmoid sinus shadow over maxillary sinus shadow on CT scans. The percentage of eosinophils in peripheral blood was also included in the score. ECRS was defined as a JESREC score of 11 or higher, in accordance with the standard cutoff (7).

### Assessment of pulmonary dysfunction

Pulmonary function tests, including forced expiratory volume in 1 s (FEV1), forced vital capacity (FVC), FEV1/FVC ratio, percentage of predicted FEV1 value (FEV1% predicted), and lower limit of normal (LLN) were performed for all participants using a standardized spirometer (Jaeger^TM^ MasterScreen^TM^ Impulse Oscillometry System, Erich Jaeger, Germany) according to the Chinese Guideline of Pulmonary Function Examination (18). Pulmonary dysfunction was defined as follows: Obstructive dysfunction: FEV1/FVC ratio below the lower limit of normal (LLN); Restrictive dysfunction: FEV1/FVC ratio ≥ LLN and FVC < LLN; Normal pulmonary function: Both FEV1/FVC and FVC ≥ LLN. A standardized measurement protocol was implemented with 30-second intervals. Following data integration, all results were evaluated by trained medical technicians, with the final approval residing with experts. Participants with either obstructive or restrictive patterns were categorized into the “pulmonary dysfunction” group for the primary analysis, while subtype analyses were also conducted.

### Clinical data collection and covariates

Covariates were based on the confounder-control strategy and the factors considered in previous studies (11–13, 15). Specifically, demographic and clinical data, including age, gender, smoking status, alcohol intake, and duration of CRS, were collected through face-to-face interviews and medical record reviews by medical staff. Height and weight were measured to calculate body mass index (BMI, kg/m^2^). Systolic blood pressure (SBP), diastolic blood pressure (DBP), fasting blood glucose (FBG), and serum lipidomic profiles, including total cholesterol (TC), triglycerides (TG), high-density lipoprotein cholesterol (HDL-C), and low-density lipoprotein cholesterol (LDL-C) were gathered based on the electronic medical record and system. Venous blood samples, taken after a more than 8-hour fast, were used to measure levels of the above biochemical examinations in the central clinical laboratory of Third Affiliated Hospital of Soochow University.

### Statistical analysis

Descriptive statistics were presented as mean ± standard deviation (SD) for continuous variables and as numbers (percentages) for categorical variables. Group comparisons separated by pulmonary function were performed using Student's t-test, ANOVA, or the Chi-square test, as appropriate. Multivariate logistic regression analyses were employed to identify independent factors associated with pulmonary dysfunction, including obstructive or restrictive patterns. For each exposure variable of interest (i.e., each individual component of the JESREC score, the total JESREC score, and ECRS), a separate multivariate model was constructed, adjusting for the same set of covariates. This approach allowed us to assess the associative strength of each component without introducing multicollinearity, given that the JESREC score inherently incorporates several of these components. Two models were constructed:

Model 1: Adjusted for age, gender, duration of CRS, smoking status, and alcohol intake.

Model 2: Further adjusted for BMI, SBP, DBP, FBG, TC, TG, HDL-C, and LDL-C.

The results are presented as odds ratios (ORs) with 95% confidence intervals (CIs). After controlling for confounding factors in the main model, mediation analysis was performed using metabolic factors that showed potential correlation with the JESREC score (continuous) and pulmonary dysfunction (BMI, SBP, DBP, FBG, TC, TG, HDL-C and LDL-C). For each candidate mediator, the exposure-mediator association (path ‘a’) was estimated using linear regression, with the JESREC score as the independent variable and the metabolic factor as the dependent variable, adjusted for the same set of covariates as in the main models. These path ‘a’ coefficients are presented in Supplementary Table 1. The significance of the indirect effect was assessed using bootstrapping with 5,000 resamples, generating a bias-corrected 95% CI. Given that eight metabolic factors were tested as candidate mediators, we applied the Benjamini-Hochberg (BH) procedure to control the false discovery rate (FDR) at 0.05. Both raw *p*-values and BH-adjusted q-values are reported for transparency. A statistically significant indirect effect was inferred if the 95% CI did not include zero. We emphasize that this statistical mediation does not imply a causal mechanism, given the cross-sectional design. In addition, the discriminative ability of the JESREC score for pulmonary dysfunction was evaluated using Receiver Operating Characteristic (ROC) curve analysis, and the area under the curve (AUC) was calculated. The optimal cutoff value was determined at the point where the sum of sensitivity and specificity was maximized. All analyses were performed using SPSS (Version 24.0) or R (Version 4.1.2), and a two-sided *P*-value < 0.05 was considered statistically significant.

## Results

### Study population and baseline characteristics

A total of 143 patients (mean [SD] age, 59.4 [11.5] years; 45.5% women; mean [SD] duration of CRS, 2.0 [3.5] years) were included in the final analysis. Based on pulmonary function tests, 56 (39.2%) patients were classified into the pulmonary dysfunction group, while 87 (60.8%) had normal pulmonary function. The general characteristics of the participants are summarized in Table 1. There were no significant differences between the two groups in terms of gender distribution, age, disease duration, smoking status, alcohol intake, BMI, blood pressure, or serum lipid profiles (all TC, TG, HDL and LDL, *P* > 0.05). However, patients with pulmonary dysfunction had significantly higher levels of fasting blood glucose (6.2 vs. 5.6 mmol/L, *P* = 0.009). As expected, all spirometric parameters (FEV1, FVC, and FEV1/FVC ratio) were significantly worse in the pulmonary dysfunction group (all *P* < 0.001). Critically, the pulmonary dysfunction group had a significantly higher JESREC score (8.6 vs. 5.0, *P* < 0.001) and a greater prevalence of ECRS (39.3% vs. 13.8%, *P* < 0.001).

**Table 1:** General characteristics of participants.

Variables	CRS participants			*P* value
	Total	With pulmonary dysfunction	With normal pulmonary function	
Male/female	78/65	35/21	43/44	0.127
Age of visit, years	59.4 ± 11.5	61.0 ± 11.4	58.4 ± 11.6	0.193
Duration of onset, years	2.0 ± 3.5	2.0 ± 2.6	2.0 ± 4.0	0.985
Smoking	15 (10.5%)	7 (12.5%)	8 (9.2%)	0.532
Drinking	13 (9.1%)	6 (10.7%)	7 (8.0%)	0.591
Body mass index, kg/m^2^	24.1 ± 3.2	24.6 ± 3.4	23.8 ± 3.0	0.137
SBP, mmHg	134.2 ± 16.9	137.0 ± 14.7	132.3 ± 18.0	0.105
DBP, mmHg	85 ± 9.2	85.2 ± 9.5	84.8 ± 9.0	0.806
Fasting blood glucose, mmol/L	5.8 ± 1.3	6.2 ± 1.6	5.6 ± 1.0	0.009*
TC, mmol/L	5.0 ± 1.1	5.0 ± 1.1	5.0 ± 1.1	0.918
TG, mmol/L	1.5 ± 0.8	1.6 ± 0.9	1.4 ± 0.8	0.107
HDL, mmol/L	1.3 ± 0.3	1.3 ± 0.3	1.3 ± 0.3	0.236
LDL, mmol/L	2.9 ± 0.9	2.9 ± 0.9	2.9 ± 0.8	0.815
FEV1, L	2.5 ± 0.6	2.5 ± 0.6	3.0 ± 0.8	<0.001*
FEV1% predicted	91.2 ± 15.1	78.0 ± 10.1	99.6 ± 11.3	<0.001*
FVC, L	2.8 ± 0.7	2.1 ± 0.4	2.7 ± 0.6	<0.001*
FVC % predicted	85.1 ± 12.9	78.0 ± 10.1	99.6 ± 11.3	<0.001*
FEV1/FVC %	87.2 ± 8.1	83.9 ± 10.8	89.3 ± 4.8	<0.001*
JESREC score	6.4 ± 4.5	8.6 ± 4.6	5.0 ± 3.9	<0.001*
ECRS	34 (23.8%)	22 (39.3%)	12 (13.8%)	<0.001*

Values are expressed as mean ± SE or *n* (%).

SBP, systolic blood pressure; DBP, diastolic blood pressure; TG, Triglycerides; HDL, High-density lipoprotein; FEV1, forced expiratory volume in 1 s; FVC, forced vital capacity; MS, metabolic syndrome.

*Significant at *p* < 0.05.

### Multivariate logistic regression for pulmonary dysfunction

Associations of JESREC score and its components with overall pulmonary dysfunction are presented in Table 2. After adjusting for covariates in model 1, bilateral lesion (OR, 3.08, 95% CI, 1.25–7.58), peripheral blood eosinophil (count, 1.26, 1.04–1.53; percentage, 1.16, 1.04–1.30), JESREC score (1.21, 1.11–1.33), and ECRS (4.08, 1.77–9.40) were identified as significant associated factors, while there was no correlation between nasal polyps, CT shadow and pulmonary dysfunction (1.58, 0.79–3.16; 1.69, 0.80–3.53), compared with the reference group. After further adjustment for BMI, blood pressure, fasting blood glucose, and serum lipids (Model 2), these associations remained robust and significant. In addition, Table 3 further shows the associations of JESREC score and its components with subtypes of pulmonary dysfunction. When the outcome was restrictive pulmonary dysfunction, results of all components were not significant, but JESREC score (1.22, 1.11–1.33) and ECRS (5.68, 2.33–13.85) were still significantly correlated with restrictive pulmonary insufficiency. When the outcome was obstructive pulmonary dysfunction, the trend was similar.

**Table 2:** Multivariate logistic regression analysis with pulmonary dysfunction as dependent variable in CRS patients.

Variables	Model 1	*P* value	Model 2	*P* value
Bilateral lesion	3.08 (1.25–7.58)	0.014*	2.90 (1.11–7.57)	0.030*
Nasal polyps	1.58 (0.79–3.16)	0.200	1.58 (0.75–3.34)	0.233
CT shadow: ethmoid ≥ maxillary	1.69 (0.80–3.53)	0.167	1.74 (0.77–3.95)	0.185
Peripheral blood				
Eosinophil, count (per 0.1 × 10^9^/L)	1.26 (1.04–1.53)	0.020*	1.30 (1.06–1.39)	0.014*
Eosinophil, %	1.16 (1.04–1.30)	0.010*	1.18 (1.05–1.33)	0.005*
JESREC score	1.21 (1.11–1.33)	<0.001*	1.24 (1.12–1.37)	<0.001*
Eosinophilic chronic rhinosinusitis	4.08 (1.77–9.40)	<0.001*	5.17 (2.05–13.09)	<0.001*

Values are expressed as odds ratio (95% Confidence interval). Each variable listed in the rows was examined in a separate multivariate logistic regression model and all models were adjusted for the same set of covariates (Model 1 and Model 2). Variables were not mutually adjusted within a single model. *Significant at *p* < 0.05.

Model 1: Adjusted for age and gender, duration of CRS, smoking status and alcohol intake.

Model 2: Further adjusted for BMI, BP, fasting blood glucose and serum lipidomic profiles.

**Table 3:** Associations of JESREC score and its components with subtypes of pulmonary dysfunction.

Outcome and Exposure	Model 1	*P* value	Model 2	*P* value
Restrictive pulmonary dysfunction				
Bilateral lesion	2.29 (0.91–5.75)	0.077	2.18 (0.80–5.95)	0.130
Nasal polyps	1.58 (0.74–3.35)	0.234	1.54 (0.67–3.52)	0.310
CT shadow: ethmoid ≥ maxillary	0.81 (0.37–1.77)	0.588	0.80 (0.34–1.89)	0.613
Peripheral blood				
Eosinophil, count	0.55 (0.13–2.29)	0.410	0.63 (0.16–2.44)	0.505
Eosinophil, %	0.96 (0.88–1.05)	0.411	0.98 (0.89–1.06)	0.565
JESREC score	1.22 (1.11–1.33)	<0.001*	1.24 (1.11–1.38)	<0.001*
Eosinophilic chronic rhinosinusitis	5.68 (2.33–13.85)	<0.001*	6.80 (2.45–18.89)	<0.001*
Obstructive pulmonary dysfunction				
Bilateral lesion	1.20 (0.15–9.39)	0.859	1.37 (0.14–13.01)	0.785
Nasal polyps	2.03 (0.70–5.87)	0.191	2.10 (0.63–7.04)	0.229
CT shadow: ethmoid ≥ maxillary	0.47 (0.15–1.45)	0.189	0.37 (0.11–1.25)	0.111
Peripheral blood				
Eosinophil, count	0.06 (0.00–3.76)	0.184	0.05 (0.00–4.58)	0.197
Eosinophil, %	0.83 (0.65–1.06)	0.138	0.82 (0.62–1.07)	0.149
JESREC score	1.23 (1.08–1.40)	0.002*	1.29 (1.10–1.52)	0.002*
Eosinophilic chronic rhinosinusitis	4.26 (1.26–14.42)	0.020*	5.39 (1.28–22.77)	0.022*

Values are expressed as odds ratio (95% Confidence interval). Each variable listed in the rows was examined in a separate multivariate logistic regression model and all models were adjusted for the same set of covariates (Model 1 and Model 2). Variables were not mutually adjusted within a single model. *Significant at *p* < 0.05.

Model 1: Adjusted for age and gender, duration of CRS, smoking status and alcohol intake.

Model 2: Further adjusted for BMI, BP, fasting blood glucose and serum lipidomic profiles.

### Mediation analysis of metabolic factors and ROC curve analysis

**Figure 1:**
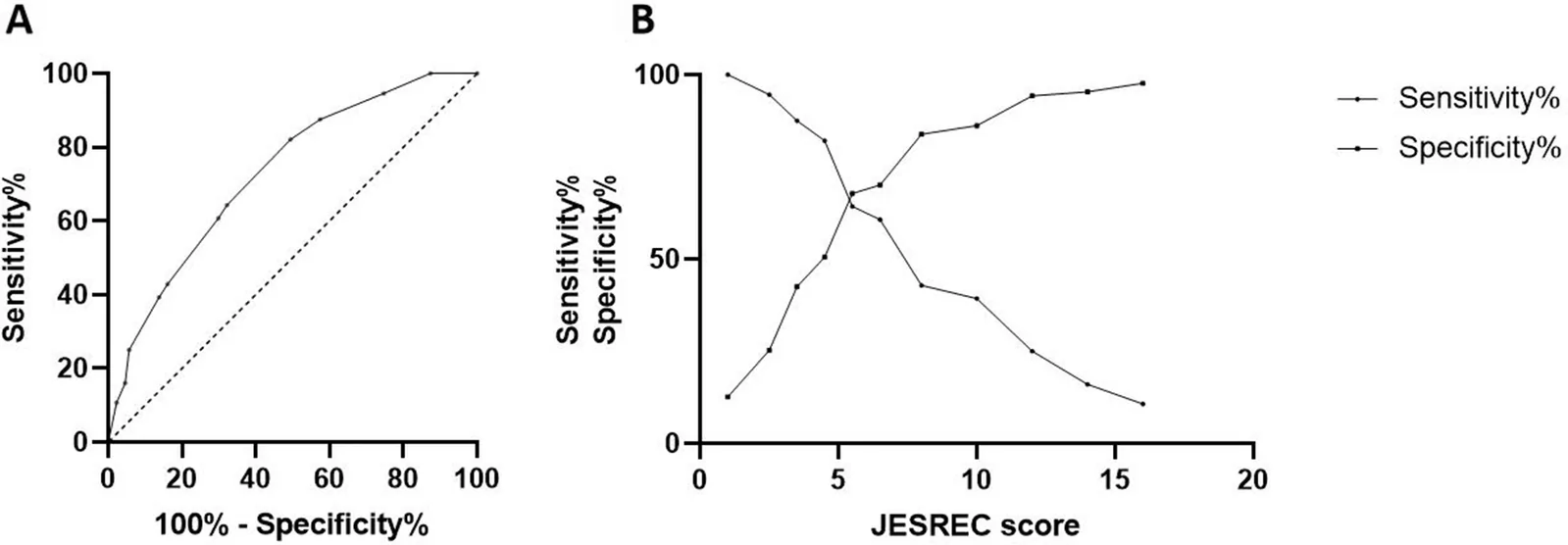
ROC curve analysis for JESREC score on pulmonary dysfunction among adult CRS patients. **(A)** ROC curve for olfactory dysfunction: AUC was 0.729. **(B)** Sensitivity-Specificity Plot: Cutoff value of JESREC score was 4.5.

**Figure 2:**
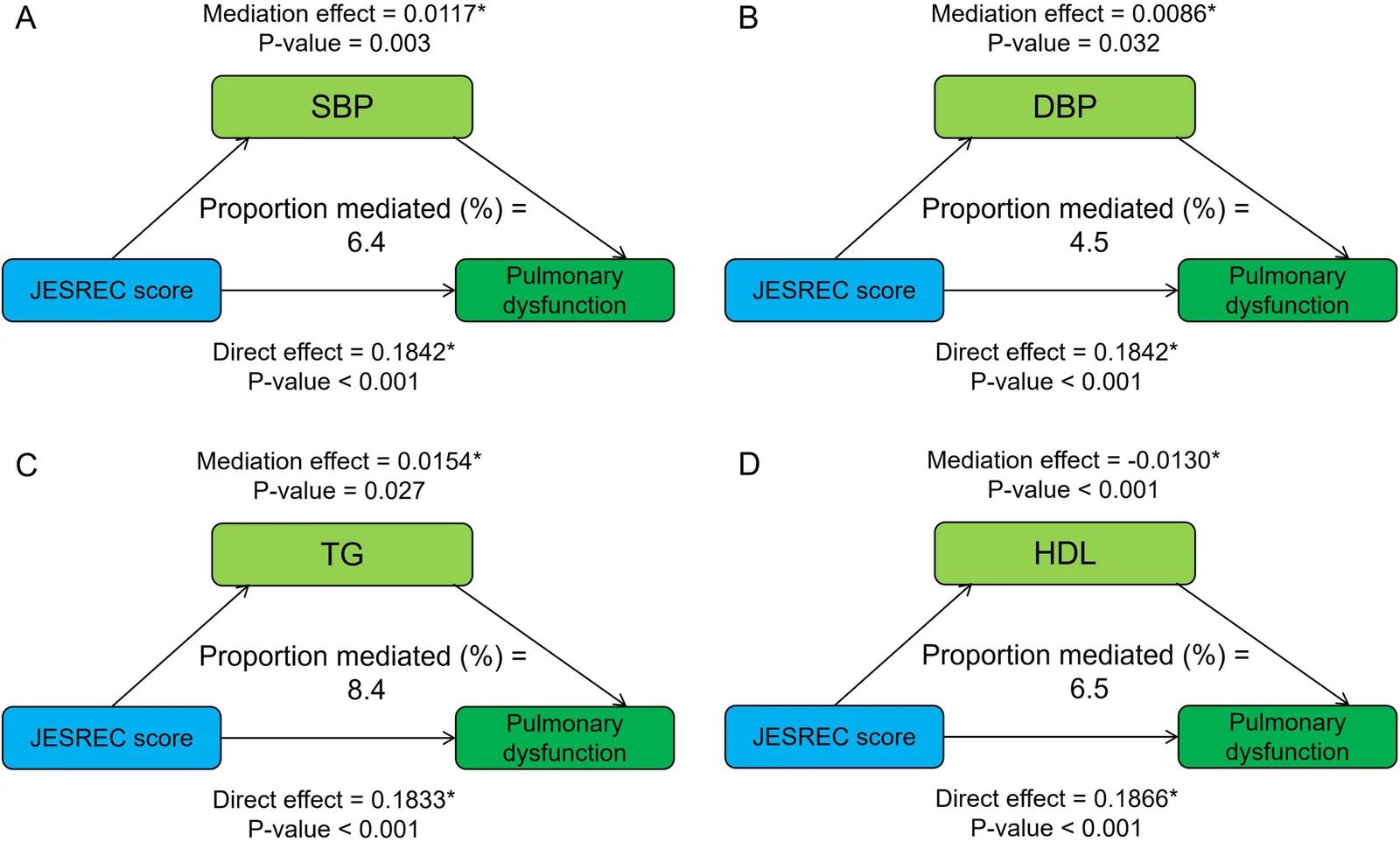
Mediation analysis of metabolic factors on the relationship between the JESREC score (continuous) and pulmonary dysfunction. *significant at *p* < 0.05.

The ROC curve analysis demonstrated that the JESREC score had a significant discriminatory ability for identifying pulmonary dysfunction among CRS patients, with an area under the curve (AUC) of 0.729 and the optimal cutoff value for the JESREC score was determined to be 4.5, which corresponded to the maximum effect value (Figure 1). In addition, Figure 2 summarizes the results of the mediation analysis examining the role of metabolic factors in the relationship between the JESREC score and pulmonary dysfunction. Specifically, SBP, DBP, TG and HDL mediated the statistical association between the JESREC score and pulmonary dysfunction at the proportions of 6.4%, 4.5%, 8.4% and 6.5%, respectively (raw *p*-values: 0.006, 0.030, 0.026, and 0.011; BH-adjusted q-values: 0.048, 0.060, 0.069, and 0.044), while the mediating effects of BMI, FBG, TC and LDL were not statistically significant (Supplementary Figure 1). Furthermore, as shown in Supplementary Table 1, the JESREC score was significantly associated with SBP (B = 0.72, 95% CI 0.18–1.35, *P* = 0.016), DBP (B = 0.47, *P* = 0.013), TG (B = 0.05, *P* = 0.010), and HDL (B = −0.02, *P* = 0.023), supporting the exposure-mediator relationships required for mediation analysis. BMI, FBG, TC, and LDL were not significantly associated with the JESREC score (all *P* > 0.05), consistent with their non-significant indirect effects.

## Discussion

This study provided a exploratory investigation into the interrelationships among eosinophilic inflammation, metabolic factors, and pulmonary dysfunction in patients with CRS. Our principal findings were threefold. First, JESREC score and ECRS were strong and independent associated with combined pulmonary dysfunction in CRS patients and this association persisted in both restrictive and obstructive of pulmonary dysfunction subtypes. Second, the JESREC score demonstrated strong discriminative ability for pulmonary dysfunction (AUC=0.729) and special attention should be paid to patients with a JESREC score ≥ 5. Third, the mediation analysis revealed for the first time that metabolic factors, including blood pressure, TG and HDL played a significant mediating role in the association between JESREC scores and pulmonary dysfunction. These findings not only strengthen the concept of “unified airway” disease, but also suggest a possible involvement of metabolic pathways, although causal inference is precluded by the cross-sectional design.

Our results strongly aligned with and extended previous studies on the upper and lower respiratory link (19–21). The strong association between JESREC score, a non-invasive alternative indicator of eosinophilic inflammation, and pulmonary dysfunction strongly suggested that a common eosinophil-oriented inflammatory process may be simultaneously invading the upper and lower respiratory tracts (22–24). Interestingly, JESREC scores were significantly associated with both obstructive and restrictive pulmonary dysfunction, which is an important finding. This indicated that eosinophilic inflammation was not only associated with asthma and other typical airway obstruction (25, 26), but may also be associated with restrictive ventilatory dysfunction—as has been observed in other eosinophilic pulmonary conditions—potentially through mechanisms that have not yet been fully elucidated, including systemic inflammatory effects on lung parenchyma or thoracic compliance (27, 28). However, we acknowledge that direct evidence for this restrictive association specifically in CRS patients remains limited, and this interpretation should be considered hypothesis-generating rather than conclusive. It should be acknowledged that in the subtype analysis, patients with pulmonary dysfunction are further divided into restrictive dysfunction and obstructive dysfunction. Given the limited sample size of each subgroup, the events-per-variable ratios are lower than the recommended threshold of 10, indicating that these analyses are exploratory in nature and should be interpreted with caution. In addition, from a clinical perspective, the strong predictive value of the JESREC score underscores its utility as a simple, clinical tool for identifying CRS patients at high risk for pulmonary dysfunction. Although the optimal cutoff was 4.5, considering that JESREC scores are all integers, we recommend that clinicians closely monitor changes in lung function in patients with JESREC scores ≥ 5.

Through mediation analysis, our study revealed the possible mechanism of metabolic factors, especially hypertension and hyperlipemia, in the “combined airway” disease. Our analysis yielded a nuanced picture: SBP, DBP, TG and HDL were identified as statistically significant mediators, while the proportions of the total effect mediated by these factors were quantitatively modest. Our study have confirmed that the systemic inflammatory state in CRS can indeed exert a measurable, albeit partial, influence on pulmonary function through its impact on specific cardiovascular and lipid metabolic pathways, however, this interpretation remains speculative and requires longitudinal validation. Furthermore, in the mediation analysis using bootstrapping (the primary method for testing indirect effects, which does not require adjustment for multiple comparisons in its original framework), SBP, DBP, TG, and HDL showed indirect effects accounting for 6.4%, 4.5%, 8.4%, and 6.5% of the total association, respectively. Nonetheless, to address concerns regarding multiple testing of eight candidate mediators, we applied the Benjamini-Hochberg procedure as a *post-hoc* sensitivity analysis. After BH correction, the adjusted q-values for DBP and TG exceeded the 0.05 threshold, which suggests that SBP and HDL may serve as more robust metabolic mediators linking eosinophilic upper airway inflammation to pulmonary dysfunction and the weaker mediation signals for DBP and TG indicate that these pathways are likely secondary and warrant further exploration. The meta-analysis results showed that patients with metabolic syndrome had restrictive rather than obstructive ventilatory dysfunction and hypertension and hyperglycemia were the core factors (29). National studies in the United States and South Korea showed that HDL-C levels were associated with improvements in FEV1 and FVC. Specifically, for each increase in HDL-C levels by one standard deviation, the predicted FVC percentage increased by 0.5% to 1.5%, and the predicted FEV1 percentage increased by 0.5% to 1.7% (30–32). In addition, mouse model showed that mice with lipid synthesis deficiency were more prone to infection and lung disease (33, 34). However, it should be emphasized that metabolic factors can only explain about 25% of the effect of CRS on pulmonary dysfunction, indicating that metabolic disorders are more of a secondary factor than a primary driver, and more research is needed to explore the possible underlying mechanisms. In addition, although the JESREC score incorporates peripheral eosinophil percentage as one of its components, it also integrates local anatomical and morphological information (bilateral lesions, nasal polyps, and CT findings) that reflect the upper airway's tissue inflammatory burden. In our separate regression models, the total JESREC score showed a higher OR compared to peripheral eosinophil percentage alone, and the ROC curve demonstrated good discriminative ability. These results suggest that the inclusion of local factors provides additional associative information beyond systemic eosinophilia alone. However, formal head-to-head comparison within a single model was not performed, and future studies are warranted to formally quantify this incremental value.

Our study has several limitations. Firstly, the single-center design with a relatively small sample size limits the generalizability of our findings. This is particularly relevant for the proposed JESREC cutoff of 4.5/≥5, which was derived from an exploratory analysis within our specific cohort. External validation in larger, multi-center prospective cohorts is therefore essential before this cutoff can be considered for clinical adoption. Secondly, cross-sectional studies cannot definitively establish the causal relationship or temporal sequence between JESREC scores, metabolic factors, and pulmonary dysfunction. While mediation analysis suggests a potential pathway, reverse causality or unmeasured confounding factors may still influence the results. Thirdly, our study focused solely on metabolic factors as mediators and future research should incorporate more comprehensive inflammatory markers and other metabolic parameters, including insulin levels and HOMA-IR to develop a more refined pathophysiological model. Furthermore, although we have adjusted for covariates and employed relatively stringent inclusion and exclusion criteria, potential confounding factors that may influence the study, such as socioeconomic bias, genetic background, and concurrent medication use (e.g., antihypertensives, statins, or inhaled corticosteroids) — all of which could directly affect the metabolic mediators (blood pressure, lipids) and eosinophil-related parameters under investigation — were not incorporated into this study. Additionally, more rigorous prospective studies are warranted to exclude conditions such as undiagnosed or subclinical asthma, which was confirmed by objective methacholine challenge or spirometric follow-up and may affect peripheral eosinophil counts and pulmonary function, as well as to further investigate the underlying mechanisms involved.

In conclusion, our study have confirmed that the JESREC score is independently associated with pulmonary dysfunction in CRS patients, and for the first time suggests a partial statistical mediation by metabolic factors. Furthermore, patients with JESREC score ≥ 5 may benefit from closer attention to pulmonary function, although external multi-center validation is needed before clinical implementation of this cutoff.

## Data Availability

The original contributions presented in the study are included in the article/Supplementary Material, further inquiries can be directed to the corresponding authors.

## Supplementary material

Supplementary Figure 1 Metabolic factors with non-significant mediating effects in the relationship between the JESREC score (continuous) and pulmonary dysfunction.
