# Supplementary material for: JESREC score, metabolic factors, and pulmonary dysfunction: analysis among adult patients with chronic rhinosinusitis
Source: Front Med (Lausanne). 2026 Sep 11;13:1859696. doi: 10.3389/fmed.2026.1859696 (PMC13612479; doi:10.3389/fmed.2026.1859696)
Supplement: Supplementary file 1 [file Table1.docx]

Supplementary Table 1. Exposure-mediator associations (path “a”) in the mediation analysis.

| Mediator | B | 95% CI | P value | R² |
| --- | --- | --- | --- | --- |
| SBP, mmHg | 0.72 | (0.18, 1.35) | 0.016* | 0.14 |
| DBP, mmHg | 0.47 | (0.09, 1.01) | 0.013* | 0.11 |
| TG, mmol/L | 0.05 | (0.01, 0.08) | 0.010* | 0.12 |
| HDL, mmol/L | −0.02 | (−0.05, −0.01) | 0.023* | 0.21 |
| BMI, kg/m² | 0.14 | (−0.09, 0.37) | 0.238 | 0.09 |
| FBG, mmol/L | 0.04 | (−0.02, 0.09) | 0.195 | 0.07 |
| TC, mmol/L | −0.03 | (−0.09, 0.04) | 0.318 | 0.04 |
| LDL, mmol/L | −0.03 | (−0.08, 0.02) | 0.334 | 0.03 |

All models were adjusted for age, gender, smoking, alcohol, and disease duration. B represents the unstandardized regression coefficient from linear regression with the JESREC score (continuous) as the independent variable and the mediator as the dependent variable. * Significant at *P* < 0.05.
